# Association between Motor Skills, Occupational Performance, and Mental Health in Japanese Children with Neurodevelopmental Disorders: A Cross-Sectional Correlational Study

**DOI:** 10.3390/children11080899

**Published:** 2024-07-26

**Authors:** Masanori Yasunaga, Hideki Miyaguchi, Chinami Ishizuki, Yosuke Kita, Akio Nakai

**Affiliations:** 1 Health and Counseling Center, Campus Life Health Support and Consultation Center, Osaka University, Toyonaka 560-0043, Japan; m.yasunaga@hacc.osaka-u.ac.jp; 2 Department of Human Behavior Science of Occupational Therapy, Graduate School of Biomedical & Health Sciences, Hiroshima University, Hiroshima 734-8551, Japan; ishizuki@hiroshima-u.ac.jp; 3 University of Kochi He alth Scienses, Kochi 781-5103, Japan; 4 Department of Psychology, Faculty of Letters, Keio University, Tokyo 108-8345, Japan; yosuke.kita@keio.jp; 5 Cognitive Brain Research Unit (CBRU), Faculty of Medicine, University of Helsinki, 00014 Helsinki, Finland; 6 Research Institute for Education & Graduate School of Clinical Education, Mukogawa Women’s University, Nishinomiya 663-8558, Japan

**Keywords:** neurodevelopmental disorder, developmental coordination disorder, executive function, motor skills, occupational performance, mental health

## Abstract

Background: Motor skills have been linked to executive functions (EFs) in children with developmental coordination disorder (DCD). However, the traits of other neurodevelopmental disorders (NDDs), such as attention-deficit/hyperactivity disorder and autism spectrum disorder, remain overlooked. Therefore, this study explored the association between motor skills, occupational performance, and mental health in older kindergarten children with DCD and other NDDs. Overall, 95 participants aged 5–6 years were included in this study and divided into four groups: DCD traits (DCD-t), DCD-t + NDD traits (DCD-t + NDD-t), NDD-t-only, and typically developing children. Motor skills, EFs, and mental health were assessed using the DCD Questionnaire (DCDQ-J) and Movement Assessment Battery for Children—Second Edition, School Assessment of Motor and Process Skills (S-AMPS), and the Strengths and Difficulties Questionnaire (SDQ), respectively. The DCD-t + NDD-t group exhibited a strong correlation between the S-AMPS motor skill score and the DCDQ-J fine motor skill score (*r* = 0.88, *p* < 0.001) and between the total DCDQ-J score and the SDQ Total Difficulties Score (*r* = −0.94, *p* < 0.001). The findings indicate that children with DCD-t and NDD-t are more likely to experience EF and mental health problems than those with DCD-t only.

## 1. Introduction

According to the *Diagnostic and Statistical Manual of Mental Disorders, Fifth Edition* (DSM-5) [1], developmental coordination disorder (DCD) is defined as markedly inferior acquisition and performance of coordination skills compared with expected skills for an individual’s age along with the opportunities to learn and use the skills. Deficiencies in motor skills lead to difficulties in activities of daily living, including infrequent participation [2], decreased self-efficacy [3], school absenteeism and bullying [4,5], and mental health disorders [6]. Therefore, early detection and intervention for children with DCD are crucial [7,8].

Previous studies have reported that 30–50% of children with attention-deficit/hyperactivity disorder (ADHD) also have DCD [9,10,11]. Children with coordination difficulties are more likely to be inattentive and hyperactive [12].

Furthermore, approximately half of children with ADHD meet the diagnostic criteria for deficits in attention, motor control, and perception (DAMP) syndrome [13], a concept reported by studies [14]. However, before the DSM-4 text revision [15], the coexistence of DCD and pervasive developmental disorder (PDD) was not recognized. However, coordination difficulties in ASD have long been recognized, and coordination disorder characteristics have been reported in approximately 80% of children with ASD [16]. To address these findings, the coexistence of DCD and autism spectrum disorder (ASD) was recognized in the DSM-5 [1]. In addition, a study using the Japanese version of the DCD Questionnaire (DCDQ-J) in Japanese boys with PDD reported that approximately 40% of the participants were clumsy, suggesting that the Autism Diagnostic Interview—Revised questionnaire correlated with the total score and subscales of the DCDQ-J [17]. Gillberg [14] found that most children diagnosed with severe DAMP syndrome have characteristics of autism, with two-thirds of the cases meeting the diagnostic criteria for ASD.

These findings indicate the necessity of closely examining the association between the development of ADHD characteristics (e.g., coordination movement, executive function (EF), reward system, and time processing) and ASD characteristics (e.g., social communication). DCD is an important characteristic of children with neurodevelopmental disorders (NDDs). Children with DCD have been reported to have EF problems [18], and their performance in EF tasks may be poorer because of primary motor deficits associated with the disorder [18,19]. EFs are divided into inhibition, shifting, and updating [20]. The evaluation of EF requires the assessment of these three components. Furthermore, children with DCD and ADHD have been reported to have significantly lower working memory (WM) scores than normal children [21,22], and they experience difficulties in planning actions and maintaining task performance [23].

Based on the ADHD characteristics [24] that coexist with DCD or the so-called DAMP syndrome and ASD [25], it was important to conduct an assessment that considers the association between coordination and EF problems [26,27] during interventions implementation [28]. Several studies have reported an association between DCD and EF issues [29,30,31,32]. However, when examining the association between DCD and EF, few studies control for comorbid disorders of DCD and examine the correlation [27,33]. In their review, Fogel et al. report that many neuropsychological assessment tools, which follow a bottom-up approach to examining EF components, may be meaningless to participants and primarily focus on dysfunction [27]. Conversely, Brown et al. argue that assessments should incorporate a top-down approach from a global perspective, emphasizing the client’s living environment [34]. Meachon et al. indicate that DCD is not merely a motor issue but is strongly associated with higher cognitive processes, such as EF [35]. Therefore, they advocate for the comprehensive assessment and treatment of DCD. Given these points, it is crucial to conduct a top-down EF assessment after distinguishing between cases of DCD alone and those with comorbid developmental disorders to investigate the relationship between DCD and EF.

According to international recommendations, activity- and participation-oriented approaches, such as Cognitive Orientation to Daily Occupational Performance (CO-OP), provide a high level of evidence for DCD interventions [11]. Although several studies have reported on interventions for children with DCD and ADHD [36,37], no study has been conducted on interventions with modified protocols based on other characteristics, such as DAMP syndrome.

Many studies have used the Performance Quality Rating Scale [38,39] and Assessment of Motor and Process Skills (AMPS) [40,41] as indicators to examine the effectiveness of interventions, with occupational performance as the primary outcome. The school version of AMPS (S-AMPS) is an assessment tool that evaluates occupational performance and is related to EF [42,43]. This performance-based assessment tool is used to observe children in places where they spend their daily lives, such as kindergartens and elementary schools. The S-AMPS allows for a detailed evaluation of children’s daily life situations and occupational performance (a part of EF) in preschool activities, making it viable for evaluating the EF of children with DCD at the activity/participation level [28]. Therefore, the S-AMPS was considered as one of the options for measuring EF in a performance-based manner.

Therefore, this study aimed to investigate the association between motor skills assessed using the DCDQ-J and Movement Assessment Battery for Children—Second Edition (MABC-2), occupational performance evaluated using the S-AMPS, and mental health assessed using the Strengths and Difficulties Questionnaire (SDQ) in older kindergarten children. Furthermore, this study focused on older kindergarten children with only DCD traits (DCD-t) and with DCD-t and NDD traits (NDD-t) to confirm the differences in the correlation between motor skills, EF, and occupational performance related to mental health, while also examining the need for a comprehensive assessment of children with DCD. The significance of this study is to clarify the association between motor skills and EF. This will contribute to developing a comprehensive assessment of each characteristic of the comorbid disorders in DCD and to tailor intervention strategies based on individual disability characteristics.

## 2. Materials and Methods

### 2.1. Participants

In this cross-sectional study, children aged 5–6 years attending certified childcare centers in the Kyushu and Chugoku regions of Japan were recruited for approximately 2 weeks in December 2015 and January 2017.

**Figure 1:**
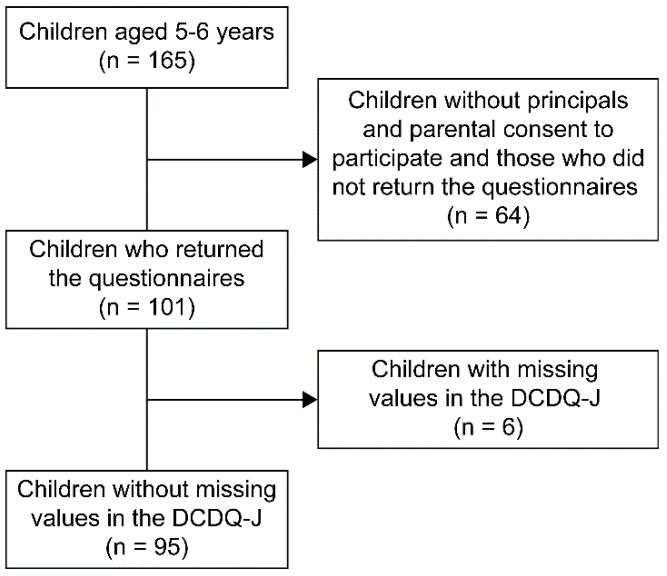
Flow chart of survey data collection. DCDQ, Developmental Coordination Disorder Questionnaire.

Out of 165 children, 95 participated in the study (mean age 75.80 ± 3.64 months, 42 boys and 53 girls). The study was conducted with the consent of the parents and the childcare centers. Certified childcare centers in Japan follow the national curriculum. Although the socioeconomic status of the parents, such as education and income levels, was not surveyed, the study focused on children from middle-class households where the impact of parental educational deficiencies and financial problems was minimal. The selection criteria included children aged 5 to 6 years who were enrolled in the certified childcare centers, with the agreement of the kindergarten principals and consent from the parents. The exclusion criteria were children with severe intellectual or physical disabilities that made communication with others extremely difficult, as verified by the teachers. Figure 1 shows a flowchart of the data collection process.

We filtered the target population to children aged 5 and 6 years because it has been reported [7] that children with DCD need to receive early intervention, that the children must understand all assessments that will be performed, and that 5 years of age is the appropriate age to start understanding the characteristics of clumsiness. This is also because the DCDQ-J targets children aged 5 years and over. The definition of DCD-t in this study was based on data from 5-year-old children obtained by Nakai et al. [44], with a total score of 40 points or less on the DCDQ-J.

The sample size was calculated using G*Power 3.1.9.7 [45] with a two-tailed test, an effect size of 0.3, a significance level of 0.05, and a power of 0.8. Consequently, the minimum sample size required was 90, assuming a dropout rate of 10%.

### 2.2. Ethical Considerations

This study was approved by the Epidemiology Research Ethics Review Committee of Hiroshima University (no. E-761) and conducted in accordance with the principles of the Declaration of Helsinki. Written informed consent was obtained from the centers’ principals, head teachers, and homeroom teachers upon written and verbal explanations. Additionally, consent was obtained from the parents after written and verbal explanations by the homeroom teachers. Participants’ questionnaires (DCDQ and SDQ) and test results (S-AMPS and M-ABC2) were stored in a locked locker, accessible only to researchers. Parents were informed in writing that they could request access to their child’s results.

### 2.3. Instruments

#### 2.3.1. Developmental Coordination Disorder Questionnaire

The DCDQ-J was developed and standardized through collaboration between Nakai et al. [44] and Wilson et al. [46], the creators of the DCDQ from Alberta Health Services and the University of Calgary, Canada, according to international guidelines [11]. The DCDQ-J is a tool used to assess children aged 5–15 years and comprises 15 items covering 3 subscales: “control during movement” (6 items), “fine motor (FM)” (4 items), and “general coordination” (5 items). Parents were asked to rate each item on a five-point scale for their children compared with others of the same age. The total scores range from 15 to 75, with lower scores indicating increasingly severe motor coordination problems. A developmental motor coordination disorder is diagnosed if the total score is below the fifth percentile and is suspected if it is between the sixth and fifteenth percentiles. The reliability, validity, high sensitivity, and specificity of the DCDQ-J have been confirmed [46,47]. Cronbach’s alpha for the DCDQ is 0.94 [48]; for the DCDQ-J subscales, the coefficients are 0.91 for control during movement, 0.91 for fine motor/handwriting, and 0.81 for general coordination [49].

#### 2.3.2. Strengths and Difficulties Questionnaire

The SDQ is a questionnaire developed by Goodman [50] for behavioral screening from infancy to school age and can assess participants’ strengths and weaknesses. Furthermore, the SDQ can be used to understand trends in peer relationships based on children’s behavioral and emotional aspects, which can facilitate reflections on their daily lives. The SDQ includes 4 subscales related to difficulties (conduct problems, hyperactivity/inattention, emotionality, and peer relationship problems) and one subscale related to strength (prosocial behavior), with each subscale comprised of 5 items (25 items). Childcare workers, teachers, and parents rate each item on a three-point scale, with 0, 1, and 2 points indicating “not applicable”, “somewhat true”, and “applicable”, respectively [48]. The need for support was evaluated on three levels based on the total score for each subcategory: high need, some need, and low need. The Total Difficulties Score (TDS) was calculated from the total score of the four difficulty-related subscales.

Based on the data on 5-year-old children from Iida et al. [51] and the Japanese version of the teacher SDQ [52], the standard value corresponding to NDD-t was the 16th percentile from the average of each sex. The TDS was 14 (9) or higher, the prosocial behavior score was 3 (5) or higher, the conduct problems score was 4 (3) or higher, the hyperactivity/inattention score was 6 (4) or higher, the emotional problems score was 4 (3) or higher, and the peer problems score was 3 (2) or lower. In this study, NDD-t was classified based on scores within the 16th percentile of the TDS or any of its subitems.

Cronbach’s alpha coefficients for the SDQ subscales are as follows: 0.83 for TDS, 0.70 for conduct problems, 0.81 for hyperactivity/inattention, 0.74 for emotion symptoms, 0.67 for peer problems, and 0.84 for prosocial behavior [51].

#### 2.3.3. School Assessment of Motor and Process Skills

The S-AMPS is the only assessment tool designed and standardized to assess the quality of students’ occupational performance [53]. This tool does not focus on diagnosis, sociability, or physical, cognitive, and psychological functions but assesses the quality of occupational performance based on motor and process skills. Motor skills pertain to physical effort, whereas process skills relate to efficiency during occupational performance [54].

The S-AMPS is comprised of 16 motor and 20 process skill items. Each item is scored on a four-point scale and represents the quality of the smallest observable units of occupational performance in the form of observable goal-oriented activities. This tool could be applied to children aged ≥3 years. The cutoff value was set at 2.0 logit for motor skills and 1.0 logit for process skills, which indicates the adaptation of the assessed child to the classroom task. The reliability and validity of the S-AMPS have been confirmed [55]. Cronbach’s alpha of the S-AMPS is 0.7 [56].

#### 2.3.4. Movement Assessment Battery for Children—Second Edition

According to international guidelines, the MABC-2 is recommended as a diagnostic tool for pediatric DCD [11], and its reliability and validity have been confirmed [57]. This tool consists of eight tasks encompassing fine motor skills and gross motor skills, organized into three subcategories: manual dexterity (three tasks: pointing coins, threading beads, and a drawing trial), aiming and catching (two tasks: catching a beanbag and throwing a beanbag onto a mat), and balance (three tasks: one-leg Balance, walking with heels raised, and jumping on mats). It consists of eight tasks, including fine and gross motor skills, and three subcategories of subtests: manual dexterity, aiming and catching, and balance. In addition to the total score, this test can calculate standardized scores for each sub-item based on performance speed and other factors.

The 5th and 15th percentiles were set as the cut-off values. Children who fall below the fifth percentile are deemed to have a serious motor function disorder, whereas those who fall between the sixth and fifteenth percentiles are deemed to be at risk of a motor function disorder [58]. Since the standardization of the Japanese version of the M-ABC2 [59,60] is still in progress, we employed original data from the United Kingdom for the calculations [57]. Cronbach’s alpha of the MABC-2 is 0.6 [59].

### 2.4. Assessment Method

The S-AMPS and MABC-2 were administered by two occupational therapists with more than 10 years of clinical experience who attended a workshop on the S-AMPS held in Japan and obtained the necessary license and participated in a workshop on the MABC-2 led by Akio Nakai and Yosuke Kita. The DCDQ-J and SDQ were administered by homeroom teachers after receiving instructions on the assessment methods.

#### 2.4.1. Implementation of DCDQ-J and SDQ

Each question in the DCDQ-J includes activities commonly seen in Japanese kindergartens. Thus, it is easy for teachers to check by recalling their usual observations of children. Therefore, the DCDQ-J was used as a screening tool.

The SDQ was developed by Goodman et al. in 1997 and is used as a general measure of mental health in various countries [61]. A Japanese version of the SDQ is available and can be downloaded for nonprofit purposes. The SDQ comprises a small number of questions and can be checked in a relatively short time. Additionally, the content of the questions aligns with activities in kindergarten. Thus, it is easy for teachers who regularly observe children to use these tools.

Data were collected as previously described by Wilson et al. [46] for the DCDQ-J and Goodman [50] for the SDQ. The questionnaires were distributed to the homeroom teachers of the classes in which the participants were enrolled. The teachers were asked to recall and check their daily activities for one month in December 2015 and January 2017.

#### 2.4.2. Implementation of S-AMPS

S-AMPS requires no special equipment and can assess the quality of task performance even when different tasks are used between the initial assessment and re-evaluation. It was chosen because it allows for the selection of tasks that align with kindergarten events and seasons.

Data were collected as previously described [53]. Two certified S-AMPS evaluators (OTs) interviewed seven homeroom teachers to determine the tasks. Teachers selected two tasks that were neither too difficult nor too easy for the children. The tasks were conducted in the children’s usual classrooms (with at least five children and one teacher). The children were instructed to retrieve tools and items from their usual places. The observation period for the two tasks was approximately 40 min, conducted from December 2015 to January 2016 or from January to February 2017.

#### 2.4.3. Implementation of M-ABC2

The Japanese version of the MABC-2, which was developed based on a contract between Akio Nakai and Pearson Education Limited, was used. The MABC-2 version (AGE BAND 1) used in this study was chosen as it was suitable and familiar for Japanese children.

Data were collected as previously described [57]. The two occupational therapists conducted one-on-one tests with the participating children in a quiet and private room at the kindergarten during free time in the morning or afternoon on weekdays when there were no group activities. Each test took approximately 25 min. The evaluations were performed from December 2015 to January 2016 or from January to February 2017.

### 2.5. Statistical Analysis

Statistical analyses were conducted using SPSS version 26 for Windows (IBM Corp., Armonk, NY, USA). Descriptive statistics were calculated for all key variables, including mean, standard deviation, median, and range, providing an overview of the participants’ characteristics and primary variables under study. Moreover, many measurements did not follow a normal distribution; therefore, non-parametric analysis was used. The reliability of the four assessments (DCDQ, SDQ, S-AMPS, and M-ABC2) was calculated using Cronbach’s alpha. Normality was evaluated using the Shapiro–Wilk test for the four groups (DCD-t, NDD-t + DCD-t, NDD-t not DCD, and typically developing (TD) children). Using one-way ANOVA and the Kruskal–Wallis test, we compared the total scores and mean values of the sub-items of the DCDQ-J, SDQ, S-AMPS, and M-ABC2 in the four groups. Pairwise multiple comparisons (Bonferroni’s method) were performed for the total scores and sub-items that showed significant differences. Furthermore, Spearman’s rank correlation coefficient analysis was used to investigate the association between the total score and the sub-items of the DCDQ-J, SDQ, S-AMPS, and M-ABC2 in 95 children and the 4 groups (DCD-t, NDD-t + DCD-t, NDD-t not DCD, and TD). The significance of the results was inferred for *p* < 0.05. The sample size for the correlation analysis was determined using G*Power 3.1.9.7 [45]. A power above 0.8 was regarded as acceptable.

## 3. Results

### 3.1. Overall Results

Of the 95 children, 17 had a DCDQ-J score of ≤40 and 47 were classified as having NDD-t based on the SDQ-TDS or subitems (16th percentile of each item). The breakdown of children within the 16th percentile in the TDS and subitems was as follows: 16 for the TDS, 4 for conduct problems, 21 for hyperactivity/inattention, 10 for emotional problems, 22 for prosocial behavior, and 34 for peer problems. Based on these results, the children were divided into four groups: DCD-t (*n* = 5), NDD-t + DCD-t (*n* = 12), NDD-t-only (*n* = 35), and TD (*n* = 43).

Table 1 shows the results of the four assessments including DCDQ-J, SDQ, S-AMPS, and MABC-2 for the four groups. Cronbach’s alpha coefficients for the four tests used in this study were as follows: For the DCDQ-J subscales, the coefficients are 0.90 for control during movement, 0.95 for fine motor/handwriting, and 0.82 for general coordination. For the SDQ subscales, the coefficients are 0.90 for TDS, 0.67 for conduct problems, 0.85 for hyperactivity/inattention, 0.80 for emotion symptoms, 0.78 for peer problems, and 0.78 for prosocial behavior. The Cronbach’s alpha coefficient for the S-AMPS is 0.86, and for the MABC-2 it is 0.80.

In the DCD-t + NDD-t group, two children had a total score in the fifth percentile on the MABC-2, and three had a total score in the fifth to the sixteenth percentile. No children in the other groups had an MABC-2 total score in the fifth or sixteenth percentile. Furthermore, seven children in the DCD-t + NDD-t group and nine in the NDD-t-only group had SDQ-TDS scores in the sixteenth percentile.

### 3.2. Association between Motor Skills, Occupational Performance, and Mental Health of 95 Participants

The DCDQ-J, SDQ, S-AMPS, and MABC-2 correlation coefficients were calculated for the 95 children (Table 2). Regarding the association between motor skills and occupational performance, the DCDQ-J total score correlated weakly to moderately with the S-AMPS motor (r = 0.23, *p* = 0.028) and process skill scores (r = 0.23, *p* = 0.023). The DCDQ-J FM score correlated weakly to moderately with the S-AMPS motor (r = 0.40, *p* < 0.001) and process skill scores (r = 0.38, *p* < 0.001). Regarding the association between motor skills and mental health, the DCDQ-J FM score correlated moderately with the SDQ-TDS (r = −0.44, *p* < 0.001), SDQ conduct problems score (r = −0.48, *p* < 0.001), and SDQ peer problems score (r = −0.49, *p* < 0.001). Regarding the association between occupational performance and mental health, the S-AMPS process skill score correlated moderately with the SDQ-TDS (r = −0.43, *p* < 0.001) and hyperactivity/inattention score (r = −0.50, *p* < 0.001).

### 3.3. Association between Motor Skills, Occupational Performance, and Mental Health of Each of the Four Groups

The DCDQ-J, SDQ, S-AMPS, and M-ABC2 correlation coefficients were calculated for the four groups (DCD-t, NDD-t + DCD-t, NDD-t + not DCD-t, and TD children) (Table 3). Regarding motor skills and occupational performance, there was no correlation between occupational performance (S-AMPS), motor skills (DCDQ-J and M-ABC2), and SDQ in the DCD-t group. Hereafter, we will discuss the results pertaining to the NDD-t + DCD-t, NDD-t + not DCD-t, and TD groups, excluding the DCD-t group.

Regarding the motor skills and occupational performance of the NDD-t + DCD-t group, the DCDQ-J FM was strongly correlated with the S-AMPS motor skill (r = 0.88, *p* < 0.001) and S-AMPS process skill (r = 0.70, *p* = 0.012). Furthermore, the S-AMPS motor skills were moderate to strongly correlated with the M-ABC2 total score (r = 0.83, *p* < 0.001), MD (r = 0.77, *p* = 0.004), and Bal (r = 0.66, *p* = 0.020). The S-AMPS process skill moderately correlated with the M-ABC2 total score (r = 0.62, *p* = 0.031), MD (r = 0.64, *p* = 0.025), and Bal (r = 0.60, *p* = 0.020). Regarding motor skills and mental health, the DCDQ-J total score was strongly correlated with the SDQ-TDS (r = −0.94, *p* < 0.001), prosocial behavior (r = 0.72, *p* = 0.008), conduct problems (r = −0.79, *p* < 0.001), hyperactivity/inattention (r = −0.93, *p* < 0.001), and peer problems (r = −0.75, *p* = 0.005).

The M-ABC2 total score was strongly correlated with the SDQ TDS (r = −0.82, *p* < 0.001), the SDQ prosocial behavior (r = 0.84, *p* < 0.001), and the SDQ peer relations (r = −0.84, *p* < 0.001).

Regarding occupational performance and mental health, the S-AMPS motor skills moderately correlated with the SDQ peer problems (r = −0.65, *p* = 0.021) and prosocial behavior (r = 0.64, *p* = 0.023). Process skill was moderately correlated with the SDQ-TDS (r = −0.66, *p* = 0.019) and peer problems (r = −0.62, *p* = 0.031).

There was no correlation between motor skills and occupational performance in the NDD-t + non-DCD-t group. Regarding motor skills and mental health, there was a moderate correlation between the DCDQ-J FM and the SDQ peer problems (r = −0.46, *p* = 0.006). Regarding occupational performance and mental health, the S-AMPS process skill was moderately correlated with the SDQ-TDS (r = −0.55, *p* < 0.001) and hyperactivity/inattention (r = −0.54, *p* < 0.001).

In the TD group, there was a weak correlation between the DCDQ-J-FM and the S-AMPS process skills (r = 0.36, *p* = 0.017). Regarding motor skills and mental health, the DCDQ-J FM was moderately correlated with the SDQ-TDS (r = −0.46, *p* = 0.002), conduct problems (r = −0.43, *p* = 0.004), and hyperactivity/inattention (r = −0.42, *p* = 0.005).

Several correlations have been identified between occupational performance and mental health. The S-AMPS process skill was moderately correlated with the SDQ-TDS (r = −0.48, *p* < 0.001), hyperactivity/inattention (r = −0.49, *p* < 0.001), and peer problems (r = −0.51, *p* < 0.001).

## 4. Discussion

### 4.1. Overall Summary

This study examined the relationships between motor skills (DCDQ-J, M-ABC2), occupational performance (S-AMPS), and mental health (SDQ) in older kindergarten children. Among the 95 participants, 17 had DCD traits (DCD-t), and 47 had neurodevelopmental disorder traits (NDD-t). The children were divided into four groups, and associations among occupational performance, motor skills, and mental health were analyzed. The analysis revealed multiple moderate to high correlations in the DCD-t + NDD-t group compared to the other groups. These findings suggest that children suspected of having DCD often have comorbid NDDs or mental health issues, emphasizing the need for early and comprehensive evaluation.

### 4.2. Correlation between Occupational Performance, Motor Skills, and Mental Health

Previous studies have reported that the S-AMPS process skills can identify components of EF [42,43] and assess elements of EF from an occupational performance perspective [28]. This study showed a significant correlation between the FM skills of the DCDQ-J and the motor and process skills assessed by the S-AMPS, which are consistent with the previous studies [62,63] that showed significant correlations between DCD motor skills and EF [26] and between FM function and EF in DCD. This is because the correlation between motor skills and EF is activated in the same brain regions, including the cerebellum, prefrontal cortex, basal ganglia, and striatum [64,65]. The brain regions, including the prefrontal cortex, cerebellum and its connecting structures, basal ganglia, and striatum, are involved in cognitive and motor tasks with EF as a correlate of behavior [66,67]. Additionally, FM skills (MABC-2 MD) require speed and accuracy. Children with DCD have impaired motor skills when they perform tasks with high executive demands, such as speed and accuracy, and WM needs [68]. These findings indirectly indicate an association between motor skills and EF. Therefore, assessment of EF in addition to motor skills in children with DCD is necessary.

Furthermore, a moderate association was observed between motor skills, occupational performance, and mental health. This is because the high proportion of children with a poor prognosis based on the SDQ total score is specific to children with coordination difficulties [69]. Additionally, it is crucial to recognize the elements that can hinder early motor development [70]. Early intervention plays an important role in prevention of the negative developmental trajectories and psychosocial effects associated with DCD [71]. These findings indicate the importance of assessing motor skills, EF, and mental health as early as 5 years of age or older when DCD can be diagnosed.

### 4.3. Usefulness of the S-AMPS for DCD Assessment

The S-AMPS can evaluate WM and planning skills in group activities, which are two components of EF that are weak in children with DCD and concurrent impairments [54,72]. Additionally, the S-AMPS process skills can be observed in EF, WM, inhibition, and shifting [28]. Mayes et al. [73] reported that formal EF tests do not capture everyday difficulties or planning issues. However, the S-AMPS can provide a detailed assessment of occupational performance (a component of EF) in informal preschool activities. Considering this, we used the S-AMPS as a primary outcome alongside motor skills in this study. Previous studies have reported issues with EF in children with DCD and its association with motor skills [29,30,31,32,62,74,75]. However, these studies may not have clarified whether the children had comorbid disorders, like ADHD, that impair EF [19]. This study evaluated the utility of the S-AMPS by distinguishing the EF characteristics and neural bases in ADHD, DCD, and their overlap. Approximately 50% of children and young adults with ADHD have difficulty regulating their behavior and exhibit high emotional instability [76]. Impaired inhibitory control is a core deficit of ADHD [77,78]. Individuals with ADHD also experience planning problems, reduced cerebellar volume, hypoactivation, and decreased temporal lobe volume [79,80]. These brain abnormalities correlate with the severity of ADHD symptoms and sensorimotor integration [81]. Studies on functional brain activity indicate dysfunction in the fronto–striatal–thalamo–temporal network during inhibition tasks in children with ADHD [82,83]. Children with DCD exhibit poor performance in visuospatial/verbal WM, inhibitory control, cognitive flexibility, and planning [29,30,74]. A study by Querne et al. [84] found response inhibition dysfunction in children with DCD, suggesting issues with the brain networks related to attention and the prefrontal cortex.

Cognitive Orientation to Daily Occupational Performance intervention has been shown to improve the functional connectivity of the default mode network and the right anterior cingulate cortex in children with DCD, which is associated with improvements in motor skills [36]. Children with co-occurring DCD and ADHD demonstrate challenges in behavioral control due to ADHD, as well as negative changes in sensorimotor integration and visuospatial attention/response inhibition areas compared to TD children.

The S-AMPS provides a top-down evaluation of activities and participation, revealing several aspects of EF [42,43]. It is crucial for assessing the activity and participation levels of children with DCD-t and for evaluating EF elements from an occupational performance perspective [28]. These findings suggest that the S-AMPS is a useful tool for evaluating motor skills and a broad range of EF in children with DCD and NDD.

### 4.4. Correlation between Occupational Performance and Motor Skills of Each of the Four Groups

In this study, a significant correlation was observed between occupational performance and motor skills in the NDD-t + DCD-t group and the TD group, but no correlation was found in the other two groups. In particular, we will discuss the relationship between occupational performance and motor skills in the NDD-t + DCD-t group.

Children diagnosed with ADHD or ASD often experience challenges in executive functioning [85]. Executive functional disorders are thought to be at the core of poor academic and occupational performance, contributing to several behavioral symptoms of NDDs [18,86,87]. Inattention and impulsive behavior in ADHD and adaptive behavioral difficulties in ASD are closely related to impairments in EF [88,89]. Furthermore, attention inhibition greatly impairs EF in children with ADHD [90]. One study reported that ADHD issues lie in inhibition rather than attention-switching [91]. Inhibition of attention may impede the ability to respond to a task [20].

In contrast, deficits in cognitive flexibility, planning [92], and generativity [93] have been reported as EF declines in pure ASD. Cognitive inflexibility results in social deficits [94] and difficulty in adapting to changing events [95].

These findings indicate an association between motor skills and EF in children with DCD and in those with comorbid disorders. A weak correlation between motor skills and occupational performance was observed among the 95 participants in this study. Therefore, identifying children suspected of having DCD who also have some form of neurodevelopmental or psychiatric issues [11] might help in understanding the characteristics of motor skills and EF along with selecting an appropriate approach. The findings also indicate that children with NDD-t and DCD-t have overlapping difficulties in EF associated with ASD and ADHD compared with children with DCD-t only, indicating a correlation between motor skills and EF.

### 4.5. Correlation between Motor Skills and Mental Health and between Occupational Performance and the Mental Health of Each of the Four Groups

This study identified correlations between motor skills, mental health, and occupational performance in the NDD-t + DCD-t, NDD-t + non-DCD-t, and TD groups. The results underscore the importance of early mental health assessment alongside motor skills evaluation. We focus on the reasons for this in the DCD-t + NDD-t group, where a strong correlation was noted between mental health and motor skills or occupational performance.

The NDD-t + DCD-t group showed a high correlation between the DCDQ-J total score and the SDQ subitem scores. Sirama et al. [96] report that suspected DCD is associated with an increased risk of emotional and behavioral problems in preschool children, and that the co-occurrence of autistic traits may be important for this association. These findings are consistent with those in our study.

Additionally, Green et al. and Zeng et al. reported that nonmotor skills, such as behavioral and socialization difficulties, might be associated with reduced physical activity [97,98]. Furthermore, opportunities for motor learning ultimately decrease [96]. Additionally, poor motor skills in early childhood can lead to a loss of opportunities for active group participation [94] and mental health problems, which are secondary to motor-related problems, that emerge as children enter elementary school and begin to meet social and peer demands [99,100].

These findings imply a strong need to conduct assessments and interventions for motor skills and mental health during early childhood. In this study, S-AMPS motor skills were moderately correlated with SDQ-TDS, peer problems, and prosocial behavior, whereas process skills were moderately correlated with SDQ-TDS and peer problems.

This finding is similar to the findings of existing studies that challenges in EF skills are not only associated with academic performance [101] but also with emotional and physical well-being [102,103] and that children with multiple comorbidities tend to have impaired EF abilities [92,104]. Based on the results of three assessments for each group, this suggests that, despite biases in the sample size and results, a high correlation exists between motor skills, EF, and mental health items in the DCD-t + NDD-t group.

Gu et al. [105] emphasize the importance of focusing on motor function and EF, as difficulties in motor coordination in children with ASD can affect social communication through EF. Children in the DCD-t + NDD-t group were more likely to have EF and mental health problems than children in the DCD-t-only group. The results suggest that it is important to test EF using the S-AMPS and to assess the comorbidity of other NDDs.

### 4.6. Limitations

This study has some limitations. First, this study had a small sample size and examined only children aged 5 years. Second, the correlation between the S-AMPS score and the DCDQ-J or MABC-2 score was not investigated in other age groups.

The association between EF and motor ability might change with development as children age and mature. Therefore, further studies with a larger sample size and a wider age range are warranted to investigate this association. Third, only a small proportion of the participants exhibited “severe clumsiness”, which limits the interpretation of these results. Fourth, tests taken by parents and other individuals were not included in the EF tests. Using rating-based EF (e.g., the Behavior Rating Inventory for Executive Function [BRIEF]—Preschool [106]) and performance-based EF (e.g., the EF Task Battery) [107] assessments is important to provide a complete picture of EF in preschool children. Furthermore, using parent evaluations of EF, such as BRIEF, and performance-based measures for children with EF is crucial to gain insights into the association between motor skills and EF in preschool children [31]. Fifth, some factors, such as sex, attention, ADHD-Rating Scale score, Autism Spectrum Screening Questionnaire score, socioeconomic status, and environmental factors (parental physical activity) were not considered in the interpretation of the results.

In the future, it will be necessary to investigate the relationship between DCD and EF by grouping children based on various factors, such as age, assessment-based evaluations, gender, attention, ADHD, ASD screenings, the economic status of their parents, and exercise habits. Additionally, to increase the number of children diagnosed with these disorders, cooperation between medical institutions and educational institutions is essential.

## 5. Conclusions

This cross-sectional study investigated the relationship between motor skills, occupational performance, and mental health in children aged 5–6 years. The results showed that children with co-occurring DCD-t and NDD-t were more likely to have EF and mental health problems than those with DCD-t alone. These findings indicate the importance of testing EF using tools, such as the S-AMPS, and evaluating the comorbidities of other NDDs, such as DAMP syndrome.

## Figures and Tables

**Table 1:** Baseline comparison of the DCD-t, NDD-t + DCD-t, NDD-t-only, and TD groups.

Outcome Measure	DCD-t	NDD-t + DCD-t	NDD-t + Not DCD-t	TD	
	(n = 5)	(n = 12)	(n = 35)	(n = 43)	
	Mean	Mean (SD)	Mean	Mean	*p*-Value
	(SD)		(SD)	(SD)	
Sex	4 boys, 1 girl	5 boys, 7 girls	16 boys, 19 girls	17 boys, 26 girls	0.259 †
Age	75.40 (1.82)	74.75 (3.75)	74.91 (3.59)	76.81 (3.58)	0.070 ^‡^
D-Total	37.20 (1.64)	33.00 (8.29)	53.97 (9.58)	56.60 (9.33)	0.000 ^‡^
D-CDM	14.10 (1.52)	11.42 (3.87)	20.00 (6.12)	20.47 (5.58)	0.000 ^‡^
D-FM	9.60 (1.34)	10.5 (4.38)	16.14 (2.90)	17.51 (2.30)	0.000 ^‡^
D-GC	13.20 (2.17)	11.08 (2.81)	17.94 (3.83)	18.81 (4.01)	0.000 ^‡^
SDQ-TDS	4.0 (3.08)	11.91 (6.58)	9.86 (4.89)	2.31 (2.62)	0.000 ^‡^
SDQ-CP	0.6 (0.89)	1.83 (1.19)	1.09 (1.38)	0.37 (0.82)	0.000 ^‡^
SDQ-HI	2.0 (1.87)	4.3 (2.67)	4.0 (2.31)	1.51 (1.72)	0.000 ^‡^
SDQ-ES	2.09 (0.45)	1.58 (2.11)	1.29 (1.84)	0.05 (0.21)	0.000 ^‡^
SDQ-PP	1.2 (0.83)	4.17 (2.12)	2.71 (1.86)	0.44 (0.70)	0.000 ^‡^
SDQ-PB	6.2 (2.17)	5.58 (2.50)	5.77 (2.29)	8.28 (1.93)	0.000 ^‡^
S-Motor	2.06 (0.70)	2.38 (0.67)	2.73 (0.39)	2.76 (0.42)	0.033 ^‡^
S-Process	0.48 (0.64)	0.67 (0.63)	0.97 (0.45)	1.01 (0.44)	0.176 §
M-Total	76.80 (7.26)	64.5 (14.83)	87.57 (7.94)	88.16 (7.90)	0.000 §
M-MD	31.00 (1.87)	23.92 (7.53)	35.77 (6.65)	35.49 (6.16)	0.000 ^‡^
M-AC	15.00 (3.08)	13.17 (3.59)	18.43 (5.38)	19.21 (4.24)	0.001 ^‡^
M-Bal	30.40 (3.21)	27.5 (7.07)	33.94 (2.95)	33.47 (3.70)	0.000 ^‡^

D, Developmental Coordination Disorder Questionnaire; S, School Assessment of Motor and Process Skills; M, Movement Assessment Battery; CDM, control during movement; FM, fine motor/handwriting; GC, general coordination; SDQ, the Strengths and Difficulties Questionnaire; TDS, Total Difficulties Score; CP, conduct problems; HI, hyperactivity /inattention; ES, emotion symptoms; PP, peer problem; PB, prosocial behavior; MD, manual dexterity; AC, aiming and catching; Bal, balance; †, chi-square test for goodness of fit; ‡, Kruskal–Wallis test. §, one-way analysis of variance.

**Table 2:** Relationship between the DCDQ and SDQ and S-AMPS and M-ABC2 in children (n = 95).

	1	2	3	4	5	6	7	8	9	10	11	12	13	14	15	16
1. D-Total	―															
2. D-CDM	0.87 **	―														
3. D-FM	0.79 **	0.48 **	―													
4. D-GC	0.87 **	0.58 **	0.73 **	―												
5. SDQ-TDS	−0.29 **	−0.14	−0.44 **	−0.36 **	―											
6. SDQ-CP	−0.35 **	−0.16	−0.48 **	−0.41 **	0.70 **	―										
7. SDQ-HI	−0.21 *	−0.10	−0.32 **	−0.30 **	0.88 **	0.51 **	―									
8. SDQ-ES	−0.09	−0.02	−0.19	−0.10	0.53 **	0.23 *	0.26 *	―								
9. SDQ-PP	−0.39 **	−0.26 *	−0.49 **	−0.37 **	0.90 **	0.62 **	0.67 **	0.48 **	―							
10. SDQ-PB	0.35 **	0.23	0.41 **	0.35 **	−0.61 **	−0.56 **	−0.46 **	−0.38 **	−0.62 **	―						
11. S-Motor	0.23 *	0.04	0.40 **	0.21 *	−0.28 **	−0.26 **	−0.28 **	−0.19	−0.22 *	0.34 **	―					
12. S-Process	0.23 *	0.13	0.38 **	0.15	−0.43 **	−0.28 **	−0.50 **	−0.14	−0.39 **	0.32 **	0.63 **	―				
13. M-Total	0.54 **	0.59 **	0.31 **	0.42 **	−0.16	−0.13	−0.14	−0.16	−0.20	0.07	−0.03	0.02	―			
14. M-MD	0.57 **	0.59 **	0.33 **	0.45 **	−0.10	−0.12	−0.03	−0.18	−0.16	0.10	−0.01	−0.02	0.75 **	―		
15. M-AC	0.36 **	0.42 **	0.21 *	0.25 *	−0.17	−0.16	−0.16	−0.17	−0.21 *	0.10	0.02	0.05	0.78 **	0.33 **	―	
16. M-Bal	0.37 **	0.39 **	0.27	0.29 **	−0.13	−0.03	−0.22 *	0.03	−0.14	0.00	0.09	0.16	0.67 **	0.31 **	0.47 **	―

* *p* < 0.05. ** *p* < 0.01. D, Developmental Coordination Disorder Questionnaire; S, School Assessment of Motor and Process Skills; M, Movement Assessment Battery; CDM, control during movement; FM, fine motor/handwriting; GC, general coordination; SDQ, the Strengths and Difficulties Questionnaire; TDS, Total Difficulties Score; CP, conduct problems; HI, hyperactivity /inattention; ES, emotion symptoms; PP, peer problem; PB, prosocial behavior; MD, manual dexterity; AC, aiming and catching; Bal, balance. Colors in the table represent the strength of the correlation, with darker colors indicating stronger correlations. Orange indicates positive correlations, and green indicates negative correlations.

**Table 3:** Relationship between the DCDQ, SDQ, S-AMPS, and M-ABC2 in children in the DCD-t + NDD-t group (n = 12).

	1	2	3	4	5	6	7	8	9	10	11	12	13	14	15	16
1. D-Total	―															
2. D-CDM	0.50	―														
3. D-FM	0.72 **	0.08	―													
4. D-GC	0.70 *	0.27	0.36	―												
5. SDQ-TDS	−0.94 **	−0.45	−0.72 **	−0.53	―											
6. SDQ-CP	−0.79 **	−0.31	−0.46	−0.75 **	0.75 **	―										
7. SDQ-HI	−0.94 **	−0.55	−0.55	−0.62 *	0.92 **	0.84 **	―									
8. SDQ-ES	−0.31	−0.33	−0.45	−0.01	0.43	−0.01	0.27	―								
9. SDQ-PP	−0.75 **	−0.37	−0.72 **	−0.32	0.89 **	0.51	0.71 **	0.61 *	―							
10. SDQ-PB	0.72 **	0.19	0.73 **	0.54	−0.84 **	−0.62 *	−0.66 *	−0.61 *	−0.84 **	―						
11. S-Motor	0.55	−0.14	0.88 **	0.37	−0.53	−0.29	−0.38	−0.49	−0.65 *	0.64 *	―					
12. S-Process	0.69 *	0.04	0.70 **	0.45	−0.66 *	−0.49	−0.57	−0.04	−0.62 *	0.55	0.63 *	―				
13. M-Total	0.78 **	0.17	0.80 **	0.46	−0.82 **	−0.49	−0.68 *	−0.67 *	−0.84 **	0.84 **	0.83 **	0.62 *	―			
14. M-MD	0.72 **	0.09	0.75 **	0.33	−0.79 **	−0.51	−0.63 *	−0.42	−0.87 **	0.69 *	0.77 **	0.64 *	0.83 **	―		
15. M-AC	0.22	0.52	0.23	−0.10	−0.30	0.02	−0.31	−0.77 **	−0.44	0.31	0.26	−0.05	0.47	0.18	―	
16. M-Bal	0.74 **	0.20	0.73 **	0.82 **	−0.59 *	−0.61 *	−0.59 *	−0.21	−0.43	0.66 *	0.66 *	0.60 *	0.61 *	0.40	0.06	―

* *p* < 0.05. ** *p* < 0.01. D, Developmental Coordination Disorder Questionnaire; S, School Assessment of Motor and Process Skills; M, Movement Assessment Battery; CDM, control during movement; FM, fine motor/handwriting; GC, general coordination; SDQ, the Strengths and Difficulties Questionnaire; TDS, Total Difficulties Score; CP, conduct problems; HI, hyperactivity /inattention; ES, emotion symptoms; PP, peer problem; PB, prosocial behavior; MD, manual dexterity; AC, aiming and catching; Bal, balance. Colors in the table represent the strength of the correlation, with darker colors indicating stronger correlations. Orange indicates positive correlations, and green indicates negative correlations.

## Data Availability

The raw data are available on Figshare (https://doi.org/10.6084/m9.figshare.25460695).
